# Discriminative biogeochemical signatures of methanotrophs in different chemosynthetic habitats at an active mud volcano in the Canadian Beaufort Sea

**DOI:** 10.1038/s41598-019-53950-4

**Published:** 2019-11-26

**Authors:** Dong-Hun Lee, Yung Mi Lee, Jung-Hyun Kim, Young Keun Jin, Charles Paull, Helge Niemann, Ji-Hoon Kim, Kyung-Hoon Shin

**Affiliations:** 1 Hanyang University ERICA Campus, 15588 Ansan, South Korea; 2 KOPRI Korea Polar Research Institute, 21990 Incheon, South Korea; 3 Monterey Bay Aquarium Research Institute, Moss Landing, California USA; 4 NIOZ Royal Netherlands Institute for Sea Research, Department of Marine Microbiology and Biogeochemistry, and Utrecht University, Den Burg, The Netherlands; 5 Department of Earth Sciences, Faculty of Geosciences, Utrecht University, Utrecht, The Netherlands; 6 Korea Institute of Geoscience and Mineral Resources, Daejeon, 34132 South Korea

**Keywords:** Biogeochemistry, Environmental sciences

## Abstract

Several mud volcanoes are active in the Canadian Beaufort Sea. In this study, we investigated vertical variations in methanotrophic communities in sediments of the mud volcano MV420 (420 m water depth) by analyzing geochemical properties, microbial lipids, and nucleic acid signatures. Three push cores were collected with a remotely operated vehicle from visually discriminative habitats that were devoid of megafauna and/microbial mats (DM) to the naked eye, covered with bacterial mats (BM), or colonized by siboglinid tubeworms (ST). All MV420 sites showed the presence of aerobic methane oxidation (MOx)- and anaerobic methane oxidation (AOM)-related lipid biomarkers (4α-methyl sterols and *sn*-2-hydroxyarchaeol, respectively), which were distinctly different in comparison with a reference site at which these compounds were not detected. Lipid biomarker results were in close agreement with 16S rRNA analyses, which revealed the presence of MOx-related bacteria (*Methylococcales*) and AOM-related archaea (ANME-2 and ANME-3) at the MV420 sites. 4α-methyl sterols derived from *Methylococcales* predominated in the surface layer at the BM site, which showed a moderate methane flux (0.04 mmol cm^−2^ y^−1^), while their occurrence was limited at the DM (0.06 mmol cm^−2^ y^−1^) and ST (0.01 mmol cm^−2^ y^−1^) sites. On the other hand, ^13^C-depleted *sn*-2-hydroxyarchaeol potentially derived from ANME-2 and/or ANME-3 was abundant in down-core sediments at the ST site. Our study indicates that a niche diversification within this mud volcano system has shaped distinct methanotrophic communities due to availability of electron acceptors in association with varying degrees of methane flux and bioirrigation activity.

## Introduction

Submarine mud volcanoes (MVs) are characterized by seepage-related geomorphological features and act as windows for emission of deep subsurface hydrocarbon gases (mainly methane), fluids, and a complex mixture of sediments^1–3^. They are considered as significant geological sources of global atmospheric methane emissions^4,5^. In the atmosphere, methane is a strong greenhouse gas that is >30 times more potent than carbon dioxide^6^. Recently, a number of active MVs featuring gas hydrate deposits were discovered in the Canadian Beaufort Sea^7,8^. This marine province is expected to experience an increases of 1 to 2 °C in bottom water temperature in the near future^9^. Similar to other Arctic continental margin sediments^10–12^, bottom water temperature increase may lead to the dissociation of gas hydrate deposits^13,14^ and elevated methane emissions to the water column and, potentially, to the atmosphere where it further contributes to global warming^8,15,16^.

Submarine MVs often host dense and diverse microbial and faunal communities fueled by gases and fluids from the subsurface^17–20^. The richness of such chemosynthetic communities depends on long-term availability of electron acceptors (e.g. oxygen and sulfate) and donors (e.g. methane) in the sediments^21,22^. In this regard, microbial processes are typically dominated by aerobic and anaerobic oxidation of methane (MOx and AOM, respectively), which are performed by different clades of aerobic methanotrophic bacteria (MOB)^23^, or anaerobic methanotrophic archaea (ANMEs)^24^. ANMEs are often accompanied by sulfate-reducing bacteria (SRB)^25–27^. At highly dynamic MVs, the capacity for methane removal is often reduced because upwelling geofluids, devoid of electron acceptors, hinder influx of electron acceptors (e.g., oxygen and sulfate) from the water column^15,19^. Physical disturbances by upwelling mud and fluids at active MVs also can give rise to heterogeneous distributions of methanotrophic communities at small scales <1 m of a single MV^28,29^.

**Figure 1:**
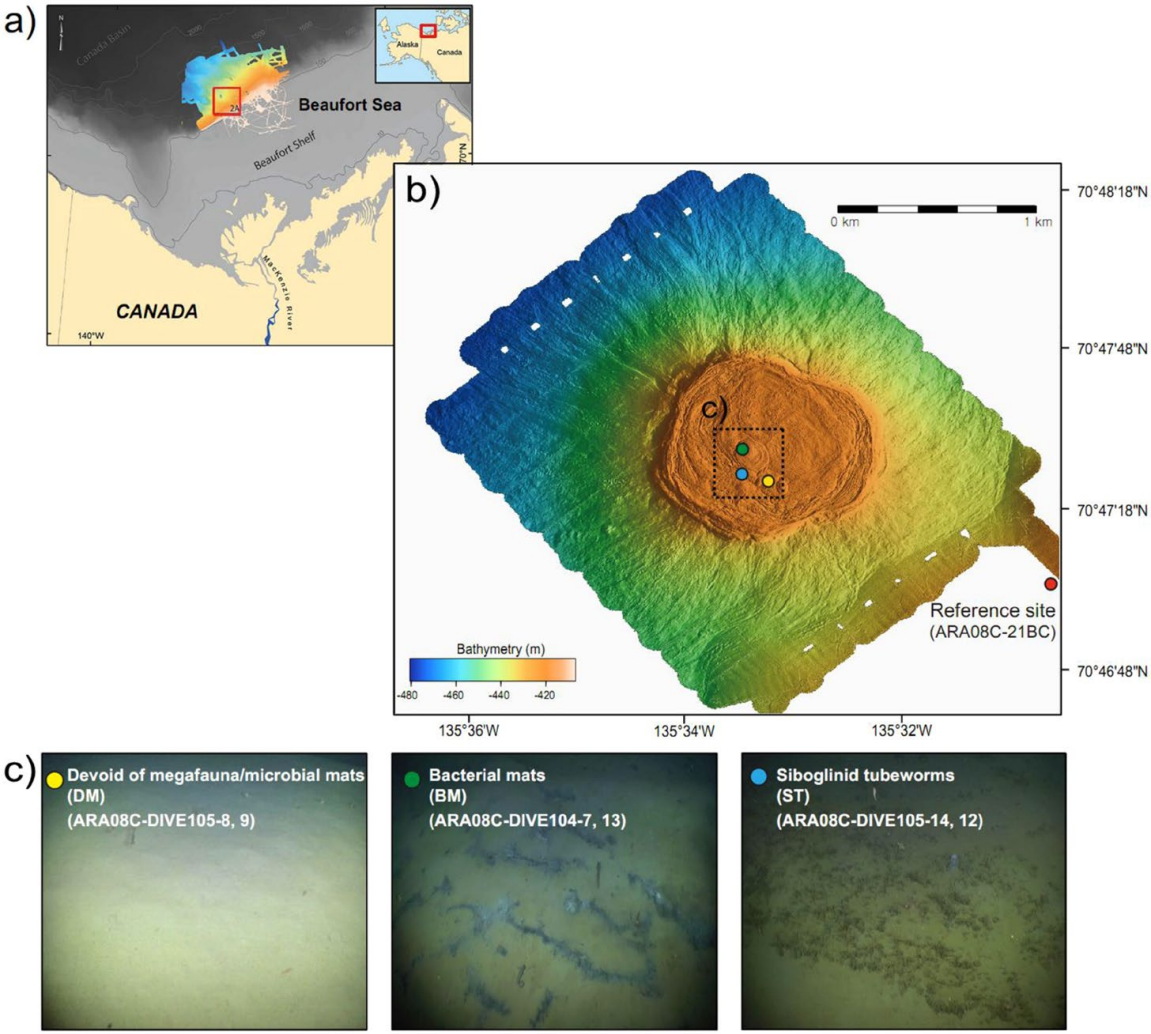
(**a**) Map showing the study area (red box) with an inset of the regional map of Alaska and northwestern Canada modified from Paull *et al*. (2015). (**b**) Map showing the mud volcano at a water depth of 420 m (MV420) and the reference site on the upper slope of the Canadian Beaufort Sea. The map was generated in Generic mapping tools version 5.4.5 (http://gmt.soest.hawaii.edu/projects/gmt). The high-resolution (1 m) bathymetry data was acquired using AUV in MBARI, processed in MB System (https://www3.mbari.org/data/mbsystem/index_ldeo.html). (**c**) Video photographs showing discriminative microbial habitats at MV420, i.e., devoid of megafauna and/microbial mats (DM), bacterial mats (BM), and siboglinid tubeworms (ST). The video photographs were taken by the MBARI ROV team during the Araon cruise in 2017.

Since the discovery of active MVs across the slope of the Canadian Beaufort Sea, the biogeochemical signatures of archaeal communities involved in AOM have shown spatially discriminative distributions among three MVs (MV282, MV420, and MV740), at water depths of 282 m, 420 m and 740 m, respectively^8,30^. In this study, we collected sediment push cores from visually discriminative chemosynthetic fields at an active MV (MV420): (i) devoid of megafauna and/microbial mats (DM) to the naked eye, (ii) covered with bacterial mats (BM), or (iii) colonized by siboglinid tubeworms (ST), using a small remotely operated vehicle (ROV) (Fig. 1). In addition, a sediment core was retrieved in a reference site, using a box corer (Fig. 1). Gas, porewater, bulk element, lipid, and nucleic acid analyses were performed to obtain a comprehensive picture of spatial variation in bacterial and archaeal methanotrophic communities. Our study of the spatial and vertical distributions of methanotrophic communities at MV420 sheds light on a key biogeochemical factor shaping discriminative communities.

## Results

### Geochemical properties

**Figure 2:**
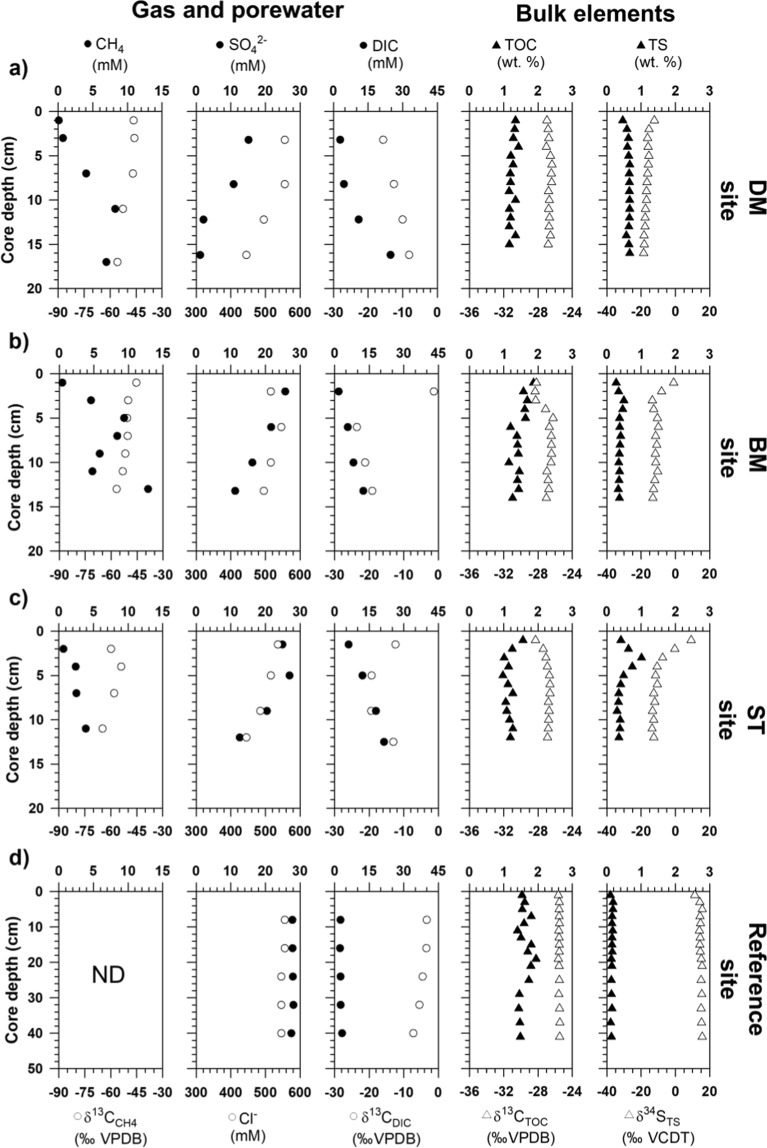
Depth profiles of gas (methane), porewater (sulfate, chloride, and dissolved inorganic carbon [DIC]), and bulk elements (total organic carbon and total sulfur) from sediment cores of (**a**) devoid of megafauna and/microbial mats (DM), (**b**) bacterial mats (BM), (**c**) siboglinid tubeworms (ST), and (**d**) reference sites. “ND” denotes “not determined.”

Methane concentration varied at three MV420 sites: DM, BM, and ST (Fig. 2). The highest methane concentration was observed at the DM site (6.6 ± 4.0 mM), followed by the BM (4.0 ± 3.6 mM) and ST (2.4 ± 1.2 mM) sites (Fig. 2). Methane concentration was below the detection limit at the reference site (Fig. 2). The carbon isotopic composition of methane (δ^13^C_CH4_) was −64.8‰ to −45.5‰, and generally showed ^13^C-enrichment toward the surface sediment layers, in accordance with the decrease of methane concentration (Fig. 2). Sulfate concentration ranged from 27.4 mM (sediment surface) to 1.1 mM (bottom section of the core), but penetration depths ranged from 1.5 cm to 7 cm, while the reference site showed a value of 27.8 ± 0.2 mM throughout the entire core (Table 1 and Fig. 2). Porewater chloride concentration varied between 495 mM and 550 mM, co-varying with sulfate concentration along the sediment depth (Fig. 2). Porewater dissolved inorganic carbon (DIC) concentration increased from surface sediments (1.8 mM) toward the bottom of the core (24.7 mM; Fig. 2). δ^13^C_DIC_ values were −11.6 ± 3.2‰ at the DM site, while those of the BM and ST site were more ^13^C-depleted (Fig. 2). In contrast, at the reference site, porewater DIC concentration was relatively constant along the sediment depth at 2.7 ± 0.3 mM, and the δ^13^C_DIC_ values were ^13^C-enriched (−4.8 ± 1.6‰). Diffusive methane flux was highest at the DM site (–648.5 mmol m^−2^ y^−1^), followed by the BM (−427.5 mmol m^−2^ y^−1^) and ST (−146.6 mmol m^−2^ y^−1^) sites (Table 1). The corresponding sulfate fluxes were 671.0 mmol m^−2^ y^−1^, 449.1 mmol m^−2^ y^−1^, and 191.4 mmol m^−2^ y^−1^, respectively.

**Table 1:** Temperature of the surface seafloor and methane and sulfate flux (calculated from concentration profiles and penetration depths of sulfate.

	Devoid of megafauna/microbial mats (DM) site (ARA08C-DIVE105-8 and 9)	Bacterial mats (BM) site (ARA08C-DIVE104-7 and 13)	Siboglinid tubeworms (ST) site (ARA08C-DIVE105-12 and 14)	Reference site (ARA08C−21BC)
Temperature (°C) (surface seafloor)	0.36	0.37	0.37	3.9
Methane flux (mmol m^−2^ y^−1^)	−648.47	−427.52	−146.56	ND
Sulfate flux (mmol m^−2^ y^−1^)	671.04	449.06	191.41	ND
Penetration sulfate depth (ca. cm)	1.5	4	7	>40

“ND” denotes “not determined.”

Total organic carbon (TOC) content ranged from 0.8 wt.% to 1.9 wt.% at all MV420 sites and from 1.4 wt.% to 1.9 wt.% at the reference site (Fig. 2). δ^13^C_TOC_ values (−28.0 ± 0.6‰) were lower in the upper 4 cm depth at the BM site, while other MV420 and reference sites showed relatively ^13^C-enriched values (−25.5 ± 0.1‰) (Fig. 2). Total sulfur (TS) content varied between 0.1 wt.% and 1.0 wt.%, with the highest value at the ST site (Fig. 2). δ^34^S_TS_ values were in the range of −18.6‰ to 9.2‰, with the largest sulfur isotopic variation (∆δ^34^S; ~20) at the ST site (Fig. 2).

### Lipid signatures of methanotrophic communities

**Figure 3:**
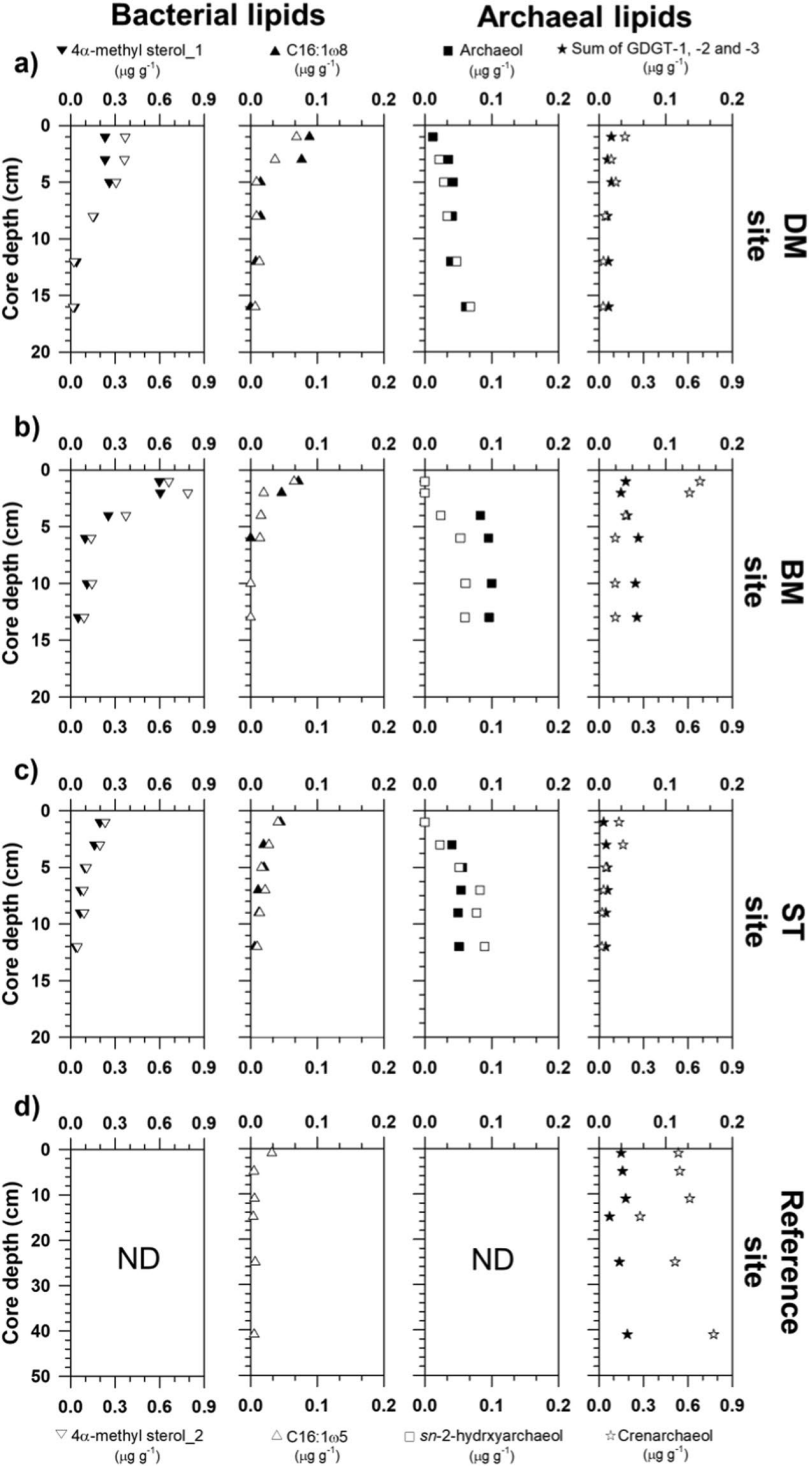
Vertical profiles of selected bacterial and archaeal lipid biomarker concentrations obtained from sediment cores of (**a**) devoid of megafauna and/microbial mats (DM), (**b**) bacterial mats (BM), (**c**) siboglinid tubeworms (ST), and (**d**) reference sites. “ND” denotes “not determined.”

The 4α-methyl sterols that are putatively specific for bacterial groups involved in MOx^31^ were abundant at the surface of all MV420 sites but not the reference site. Concentrations were highest at the surface of the BM site, with a range of 0.03–0.37 μg g^−1^ (Fig. 3 and Supplementary Information Table S1). Specific fatty acids (FAs) such as C_16:1ω8_ and C_16:1ω5_, which were inferred as markers for aerobic methanotrophic bacteria and SRB, respectively,^23,32^ were detected in a range of 0.01 to 0.14 μg g^−1^ and 0.01 to 0.09 μg g^−1^, respectively (Fig. 3 and Supplementary Information Table S1). These FAs predominated near the surface of each MV420 site and decreased along the sediment depth.

In contrast to bacterial lipids, isoprenoidal dialkyl glycerol diethers (DGDs) (e.g., archaeol and *sn*-2-hydroxyarchaeol), which are specific AOM-related biomarkers^33^, were identified in deeper sediment depths (4 to 16 cm) at each MV420 site but not at the reference site (Fig. 3 and Supplementary Information Table S1). Isoprenoid glycerol dialkyl glycerol tetraethers (GDGTs) containing zero to three cyclopentane moieties (GDGT-0 to GDGT-3) and crenarchaeol were detected in all samples investigated (Fig. 3). Isoprenoid GDGTs were dominated by GDGT-0 and crenarchaeol, at concentrations of 0.05 to 0.72 μg g^−1^ and 0.03 to 0.77 μg g^−1^, respectively, while GDGT-1, GDGT-2, and GDGT-3 were much less abundant (<0.03 μg g^−1^) in all sediments analyzed (Fig. 3 and Supplementary Information Table S1).

**Figure 4:**
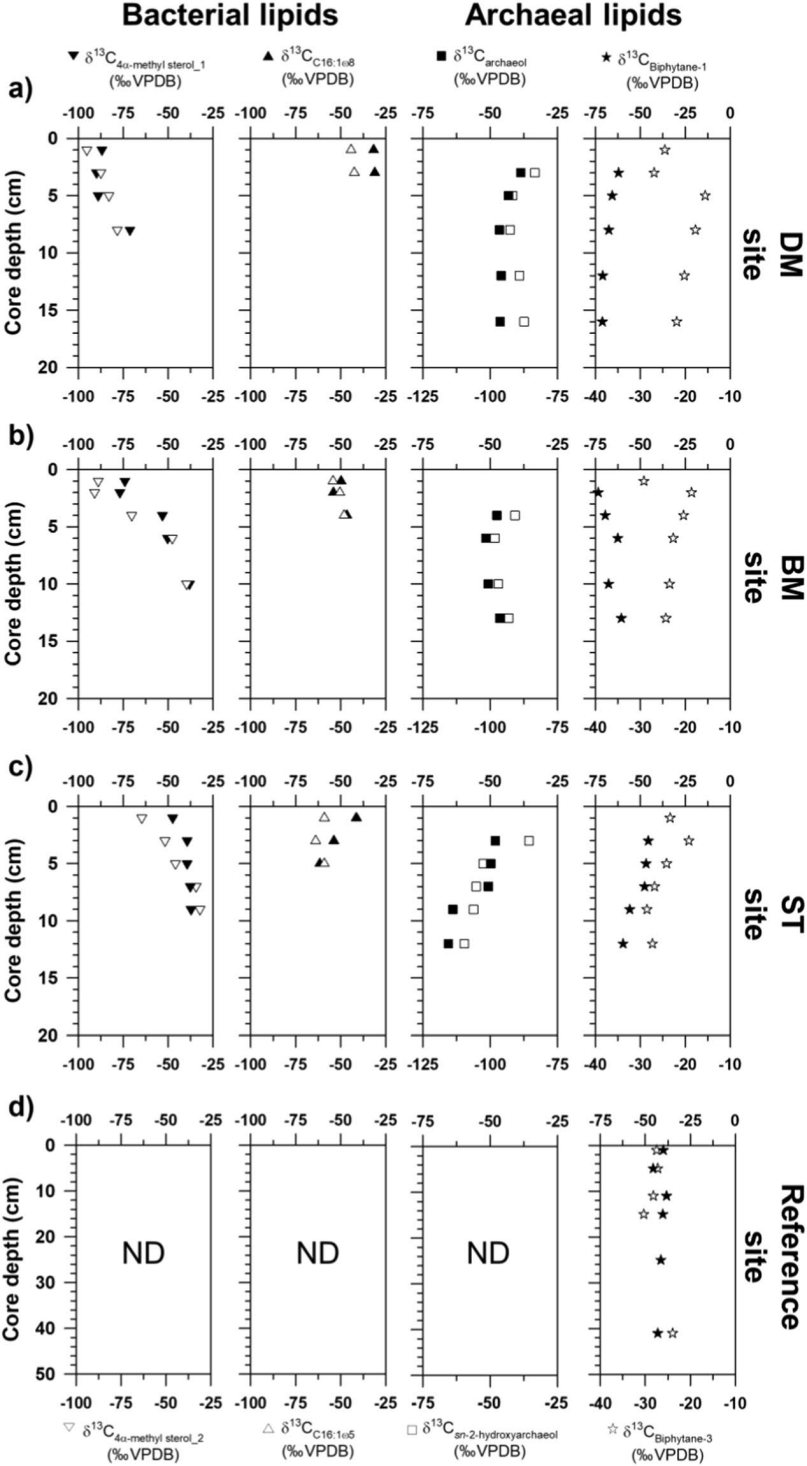
Vertical profiles of selected bacterial and archaeal lipid biomarker isotopic compositions from sediment cores of (**a**) devoid of megafauna and/microbial mats (DM), (**b**) bacterial mats (BM), (**c**) siboglinid tubeworms (ST), and (**d**) reference sites. “ND” denotes “not determined.”

The δ^13^C values of 4α-methyl sterols were −104.9‰ to −30. 5‰, and these compounds were strongly ^13^C-depleted near the surface layer of each site. The δ^13^C values of FAs ranged from −63.8‰ to −41.2‰ in all sediments investigated. The δ^13^C values of isoprenoid DGDs varied between −109.6‰ and −38.6‰, with higher depletion in *sn-*2-hydroxyarchaeol (−91.4 ± 16.4‰) than in archaeol (−47.6 ± 6.5‰) (Fig. 4 and Supplementary Information Table S1). The δ^13^C values of biphytanes (BPs) derived from isoprenoid GDGTs were in the range of −79.5‰ to −19.0‰, showing more ^13^C-depleted BP-1 values at all MV420 sites.

### Dominant methanotrophic taxa

**Figure 5:**
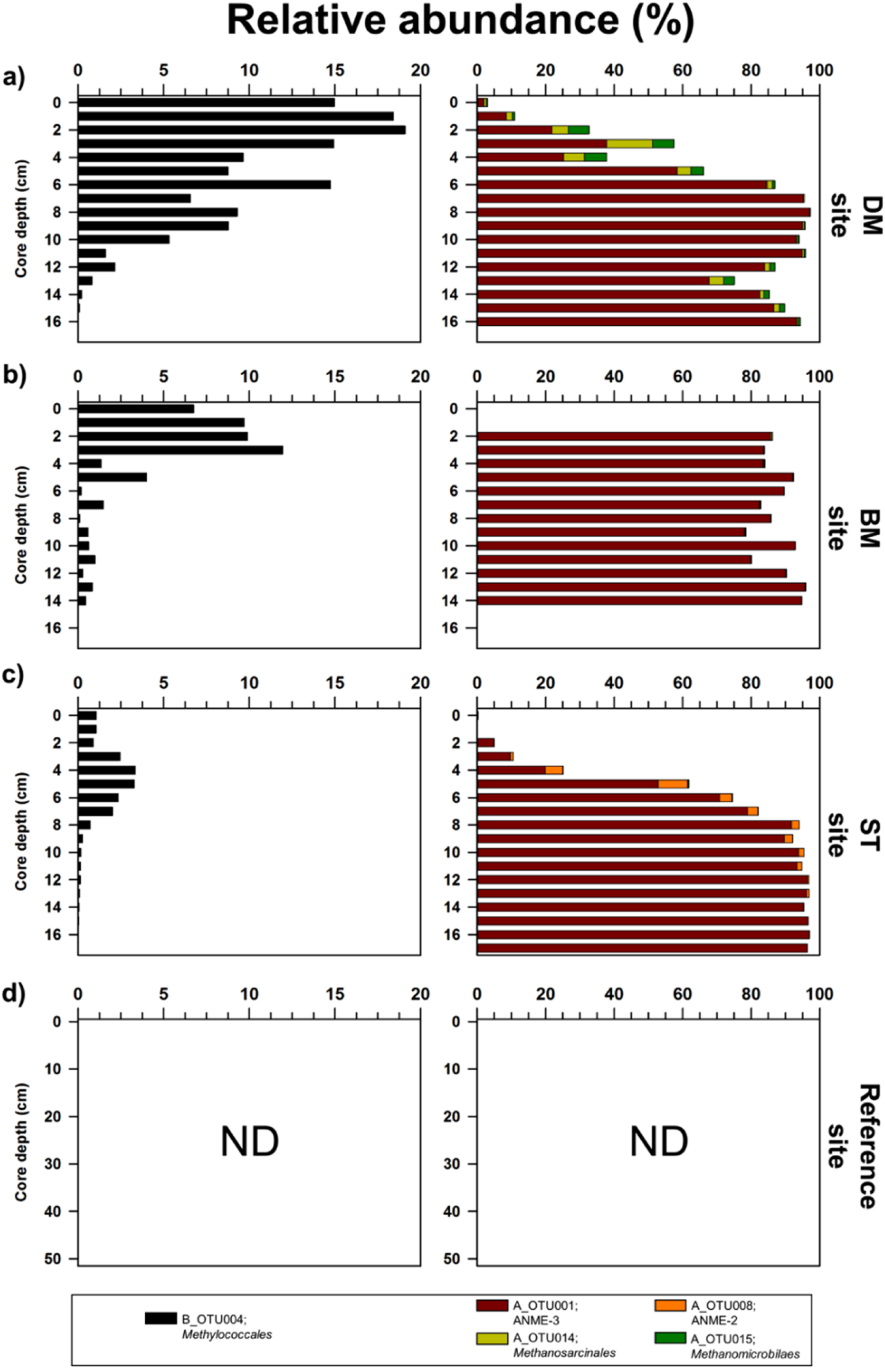
Vertical profiles of relative abundance of major OTUs (*Methylococcales*; B_OTU004 and *Methanomicrobia*; A_OTU001, A_OTU008, A_OTU014, and A_OTU015) obtained from sediment cores of (**a**) devoid of megafauna and/microbial mats (DM), (**b**) bacteria mats (BM), (**c**) siboglinid tubeworms (ST) sites, and (**d**) reference sites. “ND” denotes “not determined.”

The number of 16S rRNA gene reads of bacteria and archaea ranged from 8711 to 122,357 and from 8070 to 63,065 sequences per sample, respectively (Supplementary Information Table S2). We identified 25 operational taxonomic units (OTUs) belonging to the order *Methylococcales* of *Gammaproteobacteria*, which are aerobic methanotrophic bacteria^23,34^ (Supplementary Information Table S3 and Fig. S1). Among them, B_OTU004 dominated near the surface of the DM site (8.0 ± 6.6%), followed by the BM site (3.3 ± 4.2%) and the ST site (1.1 ± 1.2%), while it was not detected at the reference site (Fig. 5 and Supplementary Information Tables S3 and S4 and Fig. S2). With the exception of B_OTU004, the proportions of other OTUs of *Methylococcales* were less than 0.2 ± 0.3% in the samples investigated (Supplementary Information Table S3).

The relative abundance of A_OTU001, which belongs to ANME-3, was relatively higher (up to 97.1% of archaeal sequences) along sediment depths of MV420 sites, while it was less than 0.08% in the reference site (Fig. 5 and Supplementary Information Table S4 and Fig. S2). Another archaeal OTU (A_OTU008) that belongs to ANME-2 comprised 1.4% to 8.7% of archaeal sequences in the deeper sediment depths (4 to 11 cm) of the ST site. But this OTU was rare or not detected in the sediments of the DM and BM sites (Fig. 5 and Supplementary Information Table S4 and Fig. S2). A_OTU014 and A_OTU015 predominated near the surface of the DM site (up to 13.3% and 6.6% of archaeal sequences, respectively), while the proportion of these OTUs was less than 0.4% at the BM and ST sites (Fig. 5 and Supplementary Information Table S4 and Fig. S2).

Twelve OTUs of *Deltaproteobacteria* with a greater than 5% relative abundance of bacterial sequences were separately affiliated with the family *Desulfobacteraceae* or *Desulfobulbaceae* (Supplementary Information Table S4 and Fig. S2). Among eight OTUs belonging to the family *Desulfobacteraceae*, most (B_OTU002, B_OTU009, B_OTU011, B_OTU014, B_OTU024, B_OTU029, and B_OTU076) showed relatively higher abundances at deeper sediment depths at all MV420 sites but not the reference site. B_OTU014 showed the highest abundance (57.5%) at the 16 cm sediment depth of the DM site but did not exceed 2.4% at the BM and ST sites. In contrast, B_OTU018 showed the highest abundance (7.4%) near the sediment surface of the ST site. Among four OTUs belonging to the family *Desulfobulbaceae*, B_OTU010 showed relatively high abundance (2.8 to 14.4%) in the upper 3 cm of all the MV420 sites but not the reference site. In contrast, B_OTU064 showed high relative abundance only in the upper 4 cm of the ST site (2.1 to 5.5%), and the proportions of B_OTU119 was higher at the 0 to 1 cm depth of the BM site.

### Co-occurrence of ANMEs with sulfate-reducing Deltaproteobacteria

To infer the putative sulfate-reducing partners of ANMEs, co-occurrence between two archaeal OTUs (ANMEs) and 12 bacterial OTUs (*Deltaproteobacteria*) was investigated by calculating Pearson correlation coefficients (Supplementary Information Table S5). At the DM and BM sites, no OTUs showed significant correlation (*p* < 0.01) between ANMEs and SRB. At the ST site, both archaeal OTUs showed significantly positive correlation (r = 0.71 to 0.92, *p* < 0.01) with a few bacterial OTUs (B_OTU002, B_OTU011, B_OTU018, and B_OTU029).

## Discussion

### Biogeochemical characteristics along chemosynthetic habitats

Gas bubbles were observed in the ROV video rising within the water column during sampling in the chemosynthetic habitats of MV420. Previous studies using an autonomous underwater vehicle (AUV) and an ROV reported that the fresh and warm subsurface muds exposed during eruptions were mostly saturated with methane (δ^13^C_CH4_ = −64‰ and δD_CH4_ = −222‰), indicating primarily microbial sources^8,35^. Consistent with those studies, the overall geochemical signatures observed at the MV420 sites were clearly different in comparison with those of the reference site (Fig. 2).

Methane and sulfate fluxes calculated based on concentration gradient varied among the three MV420 sites (Table 1). The DM site, which was devoid of both sessile and mobile taxa such as polychaeta and bivalves, had the highest methane and sulfate fluxes (648.5 mmol m^−2^ y^−1^ and 671.0 mmol m^−2^ y^−1^, respectively). At the DM site, strong methane fluxes may cause an imbalance in hydraulic pressure, especially in shallow layers where sediments are not yet consolidated, resulting in active invasion of bottom seawater^36^. Moreover, the DM sites have been interpreted to be on mud flows which are too young to have yet recruited sessile organisms and in place not even been invaded by mobile taxa^8,37,38^. However, in accordance with decreasing fluid flux (<450 mmol m^−2^ y^−1^), free-living bacteria communities and ST patches had developed differently on the surface of MV420. Thiotrophic bacterial communities were commonly found in seeps with moderate fluid flow rates (~1 m yr^−1^), while STs dominated at seeping systems with a fluid flow rate less than 0.5 m yr^−1^ ^15,22^. Such biological succession has been reported at various seeping sites, which are differentiated by water depths and methane origins — for instance, at the Håkon Mosby mud volcano (HMMV)^19^, the Congo deep-sea fan^39^, the Gulf of Mexico^40^, and the deep Eastern Mediterranean Sea^41^. Varying fluid fluxes therefore appear to be a plausible control factor for distinguishing the near seafloor chemosynthetic habitats within MV420.

Corresponding with the depleted chloride concentrations along sediment depth profiles, the penetration depth of sulfate supplied from seawater varied among the three MV420 sites, possibly leading to isotopic variations of the total sulfur pool (Table 1 and Fig. 2). The largest isotopic variation (>20) investigated at the ST site may be attributed to the higher contributions of δ^34^S values (ca. + 20‰) from the bottom seawater sources^42^. The sulfate-methane transition zone was formed at the deeper sediment horizons of the ST site compared with other sites. In this regard, δ^13^C_CH4_ isotopic signatures were more enriched at the ST site, indicating that the remaining methane pool became progressively enriched in ^13^C^35,43,44^. These results are in close agreement with ^13^C-depleted DIC values, as a result of isotopic fractionation through active AOM under lower methane fluxes^43–46^. Considering that endosymbiotic bacteria inhabiting STs exploit some substrates (e.g., methane and by-products involved in AOM) in subsurface sediments^47,48^, bioirrigation activities by STs may enhance the availability of electron acceptors in deeper sediments^17,49–51^. These activities allow niche separation between chemosynthetic organisms competing for common energy sources over time^29,52,53^. Under varying methane fluxes investigated at MV420, both chemosynthetic organisms (e.g., free-living bacteria and STs) inhabiting seeping sediments appear to have been distinctly separated based on energy source. These results imply that different habitat types mirroring the discriminative intensities of ascending gas-fluids may influence the spatial distribution of methanotrophic communities by altering the availability of electron acceptors.

### Occurrence of aerobic methanotrophs

MOx- and AOM-related microbial communities assessed according to lipid biomarkers and 16S rRNA gene sequences showed a clear down-core shift within each site and variance among the MV420 sites. For the first time in Beaufort MV, we found abundant and ^13^C-depleted 4α-methyl sterols in concert with high proportions of OTUs belonging to the order *Methylococcale*s near the sediment surface, strongly indicating a high abundance of aerobic methanotrophs in these sediments (Figs. 3–5), as has been reported from various other seepage sites^19,28,53,54^. The predominance of aerobic methanotrophs at the surface appears to result from the intensities of oxidant-depleted subsurface fluids that limit downward diffusion of sulfate and oxygen from seawater to the uppermost centimeters of the sediment^28,53–55^. In such situations, which do not easily allow AOM reactions in deeper sediment layers, a preferential niche of MOx-related methanotrophs is formed in the surface sediment where oxygen is available. Notably, ^13^C-enriched 4α-methyl sterols in deeper sediment layers indicate that these compounds originate from processes other than methanotrophy (e.g. organisms using methanol)^56^. However, with the current data set, it is challenging to elucidate exact processes involved, and thus further research is needed.

With discriminative distributions of MOx-related methanotrophs among the three MV420 sites, ^13^C-depleted 4α-methyl sterols were much more prevalent at the BM site than at other sites (Figs. 3–5). Moreover, ^13^C-depleted TOC was abundant at the surface of the BM site, indicating that MOx-related biomass was incorporated and preserved in the organic carbon pool for this site^57–59^. Such distribution patterns investigated at MV420 appear to differ slightly from those of similar MV systems (i.e., HMMV) that showed predominant MOx-related methanotrophs under the highest methane flux^19,28^. One possible reason for this difference might be that the mud flows migrating to the tops of the DM site were too active and too young for colonization of MOx-related methanotrophs^8^, in contrast to the BM and ST sites. Moreover, previous studies of various seeping sites have suggested that seafloor temperature and oxygen availability are the key factors shaping seep microbial communities^28,54^. In this study, *in situ* bottom temperature in the surface did not vary across the MV420 sites (0.37 ± 0.1 °C), indicating that temperature is likely not the key factor explaining the discriminative distribution of MOx-related methanotrophic *Methylococcale*s within a single MV.

### Occurrence of anaerobic methanotrophs

The predominance of *sn*-2-hydroxyarchaeol over archaeol and OTUs belonging to ANME-2 and ANME-3 clades in sediment depths (4 to 16 cm) indicates the presence of AOM-related methanotrophic communities in these sediments, in contrast to the reference site (Figs. 3–5). The overall ^13^C-depleted *sn*-2-hydroxyarchaeol in all MV420 sites indicated persistent occurrence of AOM in sediments containing sulfate. Based on 16S rRNA results, ANME-3 was more dominant at the BM and ST sites than the DM site. On the other hand, ANME-2 was most frequent at the ST site, indicating a preference for a niche with lower methane flux. Notably, the low abundances of isoprenoid GDGTs (GDGT-1, GDGT-2, and GDGT-3) and 16S rRNA gene sequencing results indicate that the ANME-1 contribution was negligible for AOM in the sediments of MV420^30,33,60–62^. It is worthwhile to note that all surface sediments including that of the reference site showed a dominant crenarchaeol contribution to the total isoprenoid GDGTs pool, indicating a potential contribution of marine Thaumarchaeota^63^. The enriched δ^13^C values of archaeol and BPs suggest that archaeal communities are mixed due to processes other than AOM (e.g., assimilation of AOM-derived inorganic carbon)^64^. These δ^13^C signatures are similar to methane-related archaeal distributions investigated at other MVs (at water depths of 290 m and 740 m) in the Canadian Beaufort Sea^30^. In this regard, niche stratifications among ANMEs appear to be related to differences in environmental preferences within the sampled habitats with varying methane fluxes^53,54^.

At the three investigated MV420 sites, specific archaeal lipids and 16S rRNA signatures clearly reveal the discriminative presence of ANME-2 and ANME-3 involved in AOM between the BM and ST sites (Figs. 3–5). In contrast to ANME-1, which was observed at a wide range of temperatures (4 to 70 °C) and typically lower methane fluxes^65–67^, ANME-2 and ANME-3 have been found in samples with temperatures below 20 °C, vigorously emitting MVs such as HMMV^19,28,53,54^ and other seepages^52,66^. Among them, ANME-2 (particularly ANME-2c) was most frequently found in sediments bioturbated by benthic communities such as vesicomyid clams inhabiting low-fluid flux regimes^29,54,68,69^. Considering that vesicomyid clams have so far not been found in the western Arctic, our results indicate a requirement of ANME-2 for lower methane flux. The overall dominance of ANME-3 at the BM site is consistent with previous findings, prevailing across settings in Arctic seeping sites^19,28,53,54^. Meanwhile, at the ST site, ANME-2 can be regarded as the additional methane oxidizer under environmental conditions such as bioturbation and lower methane seepage^60,70,71^. Given that the produced sulfide is quickly oxidized by thiotrophic bioturbators, the presence of ANME-2 below tubeworm patches may imply that they seem to form a syntrophic relationship with SRB during AOM^19,54^. To some degree, ANME-3 appears to tolerate high sulfide concentrations produced during active AOM processes^72^, although the sulfide sensitivity of ANMEs needs to be investigated further. AOM activities performed by both ANMEs seem to support the metabolism of sulfur-oxidizing endosymbionts inhabiting frenulata *Oligobrachia haakonmosbiensis*^73,74^. The presence of diverse methanotrophs at the ST site can therefore be explained by environmental conditions under steady replenishment of sulfate and oxygen levels into the sediments, in accordance with the development of ST patches.

### Niche adaptation between ANMEs and SRBs

With respect to the consortium of ANMEs and SRBs, the presence of sulfate-reducing Deltaproteobacteria, including the partner SRB of ANMEs, was assessed by analyzing specific FAs and deltaproteobacterial OTUs (*Desulfobacteraceae* and *Desulfobulbaceae*) at the three MV420 sites. The ^13^C-depleted, SRB-specific lipid (C16:1ω5) probably derived from *Desulfobacteraceae* was relatively abundant near the sediment surface, while another specific FA (C17:1ω6) derived most likely from *Desulfobulbaceae* was not identified in any sediments^60^. Most deltaproteobacterial OTUs showed non-significant correlations with major OTUs of the ANME-2 and ANME-3 clade. Only a few deltaproteobacterial OTUs had a positive correlation with major ANMEs at the ST site. This suggests a weak coupling between ANMEs and *Desulfobacteraceae* at the MV420 sites, which is inconsistent with reported couplings of SRBs (*Desulfosarcina/Desulfococcus* and *Desulfobulbus*) and ANMEs (ANME-2 and ANME-3) at HMMV^19,28^. *Desulfobacteraceae* often occur as single cells in surface sediments of bacterial mat- or clam-covered seeps^75,76^ and decrease with increasing sediment depths^75^. Therefore, this SRB seems to have a metabolic advantage over other SRBs in oxygenated sediments. Accordingly, at MV420, the occurrence of overall ANMEs without partner SRBs may imply that ANMEs were either associated with other SRBs, occurred as loose aggregates or a single cell without direct contact with SRBs^24^, or performed AOM without a partner SRB^77^. More studies are necessary to further investigate ANME-SRB consortia by, for example, applying the technique of fluorescence *in situ* hybridization.

## Summary

In this study, we provide the first evidence for MOx- and AOM-related methanotrophic communities in connection with chemosynthetic communities at an active MV in the Canadian Beaufort Sea. Our results revealed that niche diversification of methanotrophic communities (i.e., *Methylococcales* and ANME-2 and ANME-3 groups, respectively) may have been shaped by environmental factors such as availability of electron acceptors and bioirrigation activities by chemosynthetic organisms. In comparison with highly local diversification of methanotrophic communities at heterogenous seeping sites^54^, our finding confirms that the supply of electron acceptors is a key factor determining local diversification of methanotrophic communities. In particular, our study is one of the few that show both methanotrophs (i.e., *Methylococcales* and ANME-3) distributed separately under seeping fluids and high methane fluxes^19,24,28,66^. Our study of methanotrophic signals provides valuable information on the effective removal pathways involved in methane release from marine sediments.

## Methods

### Sample collection

Sediment samples were obtained during the 2017 ARA08C cruise of the Korean icebreaker *RV Araon* at the Beaufort MV (water depth of 420 m) from different chemosynthetic provinces (ARA08C-DIVE105-8 and -9: BO site, ARA08C-DIVE104-7 and-13: BM site, and ARA08C_DIVE105-12 and -14: ST site) using a push core operated by an ROV system and at the reference site (ARA08C-21BC, water depth of 420 m) using a box corer (Fig. 1). Upon recovery, push- and box-core samples were subsampled horizontally with 20 mL serum vials, which were then sealed immediately with butyl rubber stoppers to prevent gas exchange and stored at 4 °C. Porewater was extracted from holes drilled at intervals of 2 to 3 cm down the core liner using rhizon samplers connected to 20 mL syringes. Extracted porewater was filtered through a 0.20 μm disposable polytetrafluorethylene in-line filter and stored at 4 °C until analysis. After porewater extraction, core sediments were sliced into 1cm sections and subsampled for analysis of bulk elements, lipid biomarkers, and 16S rRNA gene sequences. Subsamples were stored at −80 °C until analysis.

### Gas and porewater analyses

Headspace methane concentration was measured with a gas chromatograph (GC) (7890 A, Agilent Technologies, CA, USA) with flame ionization detector. The precision of repeated standard analyses exceeded 5%. Isotopic compositions of methane were obtained using an isotope ratio mass spectrometer (Finnigan MAT 252; Thermo Fisher Scientific, Waltham, MA, USA) with a Combustion III interface (Thermo Fisher Scientific) at Nagoya University. Isotopic values were expressed as δ-notation (per mil) relative to Vienna Pee-Dee Belemnite (VPDB). The δ^13^C values were calibrated using analyses of a methane standard with δ^13^C value of −35.2‰, as certified by the National Institute of Standards and Technology (NIST, USA). Precision for δ^13^C_CH4_ was greater than ±0.2‰.

Chloride (Cl^–^) and DIC were analyzed by titration using silver nitrate (AgNO_3_) and 0.1 N HCl, respectively. The precision determined by repeated titrations of International Association of Physical Sciences of the Oceans standard seawater was ±0.5% for Cl^–^ and <2% for DIC. Sulfate was measured using ion chromatography (Metrohm 761). The δ^13^C values of DIC were obtained using an isotope ratio mass spectrometer (Finnigan DELTAplusXL; Thermo Fisher Scientific, Waltham, MA, USA) with a Finnigan GasBench-II headspace autosampler at Oregon State University (Corvallis, OR, USA). Isotopic values were expressed as δ-notation (per mil) relative to VPDB. The δ^13^C values were calibrated through analyses of a CaCO_3_ standard with δ^13^C value of –0.4‰, certified by Oregon State University. The precision for δ^13^C_DIC_ was greater than ±0.02‰.

### Calculation of methane and sulfate fluxes

Assuming that related flux over the linear range of the sulfate profile was proportional to methane flux at a particular location^78^, we determined an AOM depth where the linear sulfate and methane concentration gradients overlapped in each core sediment. Diffusive fluxes of solutes (methane and sulfate) into the AOM layers were determined using Fick’s first law, and the slope of methane and sulfate concentration was plotted against depth over a linear range of data^79,80^ using the equation

$$J=-{\rm{\phi }}{D}_{s}\frac{\partial {\rm{C}}}{\partial {\rm{z}}}$$

where *J* represents the diffusive methane and sulfate fluxes (mmol m^–2^ yr^–1^), φ is the sediment porosity, *D*_*s*_ is the sediment diffusion coefficient, C is the range of methane and sulfate concentrations, and z is the range of depth for the linear section of the methane and sulfate porewater profiles. *D*_*s*_ values were calculated from free diffusion coefficients in seawater (D_0_, cm^2^ yr^–1^) by correcting for sediment tortuosity^81^:

$${D}_{s}=\frac{{D}_{0}}{1-\,\mathrm{ln}({\phi }^{2})}$$

Downward sulfate diffusion into the sediment is expressed as negative, and upward methane flux out of the sediment is expressed as positive.

### Bulk element analysis

Sediment samples were freeze-dried and homogenized using an agate mortar prior to bulk geochemical analyses. For TOC analysis, sediment samples (~1 g) were treated with 8 mL of 1 N HCl to remove carbonates. The TOC content and its isotopic composition were measured using an elemental analyzer (EuroEA3028, Eurovector, Milan, Italy) connected to an isotope ratio mass spectrometer (Isoprime, GV instruments, Manchester, UK) according to Lee *et al*. (2018). Isotopic values are expressed as δ^13^C values in per mil relative to VPDB. The δ^13^C values were calibrated through a CH_3_ standard with a δ^13^C value of −24.7‰, certified by the International Atomic Energy Agency (IAEA, Austria). The precision for TOC and δ^13^C_TOC_ was better than ±0.2 wt.%, and ±0.1‰, respectively. The total sulfur (TS) content and its isotopic composition were measured using an elemental analyzer (EA1110, Thermo) connected to an isotope ratio mass spectrometer (Dual pumped 20–20 S, Secon). Isotopic values are expressed as δ^34^S values in per mil relative to Vienna Canyon Diablo Troilite (VCDT). The δ^34^S values were calibrated using S-2 and S-3 standards with δ^34^S values of 22.7‰ and −32.3‰, respectively, certified by the IAEA. The precision for TS and δ^34^S was better than ±0.4 wt.% and ±0.4‰, respectively.

### Lipid biomarker analysis

Lipid extraction and purification for different compound classes and analyses by gas chromatography, gas chromatography–mass spectrometry, gas chromatography–combustion–isotope ratio mass spectrometry, and high-performance liquid chromatography–atmospheric pressure positive–ion chemical ionization–mass spectrometry were conducted according to previously reported methods^30,61,82^. Double-bond positions in fatty acid methyl esters were determined by analysis as their dimethyl disulfide adducts according to the method of Nichols *et al*. (1986). All isotopic values were reported using δ-notation (per mil) with respect to VPDB. The precision for δ^13^C values was greater than ±0.5‰, corrected for introduction of additional carbon atoms during sylilation and methylation.

### Genomic DNA extraction, amplification, and sequencing

Genomic DNA was extracted using an Exgene Soil SV mini kit (Cambio, UK) at GeneAll (Seoul, South Korea). The V4-V5 regions of bacterial 16S rRNA genes and V6-V8 regions of archaeal 16S rRNA genes were amplified by polymerase chain reaction using primer pairs 515F/926R and A956F/A1401R, respectively, at the Integrated Microbiome Resource (IMR) at Dalhousie University, Canada (http://cgeb-imr.ca)^83^. Sequencing of the amplicons was carried out at IMR using the paired-end (2 × 300 bp) Illumina MiSeq system (Illumina, USA).

### 16S rRNA gene sequences processing

Pair end sequences were trimmed based on Sickle^84^ quality scores followed by BayesHammer error corrections^85^. The resulting quality-trimmed and error-corrected paired end sequences were assembled using PANDAseq.^86^. Further sequence-processing steps were performed according to the mothur Pipeline package^87^. The assembled bacterial sequences were aligned against a SILVA alignment (http://www.arb-silva.de), and archaeal sequences were aligned against a SINA alignment^88^ and subsequently denoised using the “pre.cluster” command. Chimeric sequences were removed using the “chimera.uchime” command in de novo mode^89^. The sequences were further clustered to OTUs at a 97% sequence similarity level using the opticlust clustering algorithm. Taxonomic assignments of each OTU were determined by sequence similarity searches against the EzBioCloud database^90^. All sequence data used in this study were deposited in the Sequence Read Archive at the National Center for Biotechnology Information under the accession number PRJNA559003.

### Phylogenetic analysis

Phylogenetic trees of major archaeal OTUs of *Methanomicrobia,* bacterial OTUs of *Deltaproteobacteria* and *Methylococcales* of *Gammaprotoebacteria* with greater than 5% relative abundance (Supplementary Information Sequence 1–3) were constructed using the maximum-likelihood algorithm^91^ with the GTR evolutionary model using IQ-TREE^92^.

## Supplementary information

Supplementary Material

Supplementary Material

Supplementary Material
