# Supplementary material for: Discriminative biogeochemical signatures of methanotrophs in different chemosynthetic habitats at an active mud volcano in the Canadian Beaufort Sea
Source: Sci Rep. 2019 Nov 26;9:17592. doi: 10.1038/s41598-019-53950-4 (PMC6879587; doi:10.1038/s41598-019-53950-4)
Supplement: Supplementary file 1 — Supplementary Material [file 41598_2019_53950_MOESM1_ESM.docx]

**Supplementary Information**

**Discriminative biogeochemical signatures of methanotrophs inhabiting different chemosynthetic habitats in an active mud volcano of the Canadian Beaufort Sea**

Dong-Hun Lee^1,^†, Yung Mi Lee^2,^†, Jung-Hyun Kim^2,*^, Young Keun Jin^2^, Charles Paull^3^, Helge Niemann^4,5^, Ji-Hoon Kim^6^, Kyung-Hoon Shin^1,**^

^1^Hanyang University ERICA campus, 15588 Ansan, South Korea

^2^KOPRI Korea Polar Research Institute, 21990 Incheon, South Korea

^3^Monterey Bay Aquarium Research Institute, Moss Landing, California, USA

^4^NIOZ Royal Netherlands Institute for Sea Research, Department of Marine Microbiology and Biogeochemistry, and Utrecht University, NL-1790 AB Den Burg, The Netherlands

^5^Department of Earth Sciences, Faculty of Geosciences, Utrecht University, Utrecht, The Netherlands

^6^Korea Institute of Geoscience and Mineral Resources, Daejeon 34132, South Korea

†These authors contributed equally to this work.

^*^ Corresponding author:

Tel.: +82 32 760 5377

jhkim123@kopri.re.kr

^**^ Co-corresponding author:

Tel.: +82 31 400 5536

shinkh@hanyang.ac.kr

Table S1. Lipid concentrations and carbon isotopic compositions in push core sediments of MV420.

| **Core depth (cm)** | **Archaeal lipids** | | | | | | | | | | | | | **Bacterial lipids** | | | | | |
| --- | --- | --- | --- | --- | --- | --- | --- | --- | --- | --- | --- | --- | --- | --- | --- | --- | --- | --- | --- |
|  | **Archaeol** | | ***sn*-2-hydroxyarchaeol** | | **GDGT-0** | **GDGT-1** | **GDGT-2** | **GDGT-3** | **Crenarchaeol** | **Biphytane-0** | **Biphytane-1** | **Biphytane-2** | **Biphytane-3** | **4α-methyl sterols** | | **C16:1ω8** | | **C16:1ω5** | |
|  | **μg g^-1^ dw** | **‰ VPDB** | **μg g^-1^ dw** | **‰ VPDB** | **μg g^-1^ dw** | **μg g^-1^ dw** | **μg g^-1^ dw** | **μg g^-1^ dw** | **μg g^-1^ dw** | **‰ VPDB** | **‰ VPDB** | **‰ VPDB** | **‰ VPDB** | **μg g^-1^ dw** | **‰ VPDB** | **μg g^-1^ dw** | **‰ VPDB** | **μg g^-1^ dw** | **‰ VPDB** |
| **DM site** |  |  |  |  |  |  |  |  |  |  |  |  |  |  |  |  |  |  |  |
| **1** | 0.01 | ND | ND | ND | 0.15 | 0.01 | 0.01 | 0.00 | 0.18 | -33.3 | ND | -39.7 | -24.5 | 0.30 | -91.1 | 0.09 | -31.7 | 0.07 | -44.1 |
| **3** | 0.04 | -38.6 | 0.02 | -83.4 | 0.07 | 0.01 | 0.01 | 0.00 | 0.08 | -31.3 | -62.1 | -39.1 | -26.9 | 0.30 | -88.7 | 0.08 | -31.1 | 0.04 | -42.3 |
| **5** | 0.04 | -43.2 | 0.03 | -91.7 | 0.13 | 0.01 | 0.01 | 0.00 | 0.11 | -40.0 | -65.6 | -42.9 | -15.6 | 0.28 | -86.0 | 0.01 | ND | 0.01 | ND |
| **8** | 0.04 | -46.5 | 0.03 | -92.6 | 0.05 | 0.01 | 0.01 | 0.00 | 0.04 | -42.6 | -67.5 | -36.3 | -17.7 | 0.15 | -74.8 | 0.01 | ND | 0.01 | ND |
| **12** | 0.04 | -45.9 | 0.05 | -89.1 | 0.05 | 0.01 | 0.01 | 0.00 | 0.03 | -37.5 | -70.9 | -54.7 | -20.2 | 0.01 | ND | 0.01 | ND | 0.01 | ND |
| **16** | 0.06 | -46.3 | 0.07 | -87.4 | 0.05 | 0.01 | 0.01 | 0.00 | 0.03 | -32.2 | -71.1 | -27.5 | -21.9 | 0.01 | ND | ND | ND | 0.01 | ND |
|  |  |  |  |  |  |  |  |  |  |  |  |  |  |  |  |  |  |  |  |
| **BM site** |  |  |  |  |  |  |  |  |  |  |  |  |  |  |  |  |  |  |  |
| **1** | 0.00 | ND | ND | ND | 0.43 | 0.02 | 0.01 | 0.01 | 0.68 | -30.1 | ND | -36.7 | -29.2 | 0.63 | -81.5 | 0.07 | -49.7 | 0.07 | -54.1 |
| **3** | 0.00 | ND | ND | ND | 0.25 | 0.02 | 0.01 | 0.01 | 0.61 | -30.5 | -73.4 | -43.9 | -18.6 | 0.70 | -84.0 | 0.05 | -53.9 | 0.02 | -50.3 |
| **5** | 0.08 | -47.5 | 0.02 | -90.8 | 0.14 | 0.02 | 0.01 | 0.01 | 0.18 | -31.4 | -69.5 | -40.5 | -20.4 | 0.31 | -61.8 | 0.02 | -46.4 | 0.02 | -48.0 |
| **8** | 0.10 | -51.6 | 0.05 | -98.3 | 0.16 | 0.02 | 0.02 | 0.02 | 0.11 | -33.3 | -62.5 | -33.4 | -22.7 | 0.12 | -49.0 | ND | ND | 0.01 | ND |
| **12** | 0.10 | -50.7 | 0.06 | -96.9 | 0.15 | 0.02 | 0.02 | 0.01 | 0.11 | -32.7 | -67.6 | -47.6 | -23.5 | 0.13 | -38.9 | ND | ND | 0.00 | ND |
| **16** | 0.10 | -46.5 | 0.06 | -93.1 | 0.16 | 0.02 | 0.02 | 0.01 | 0.11 | -31.9 | -60.5 | -42.5 | -24.3 | 0.01 | ND | ND | ND | 0.00 | ND |
|  |  |  |  |  |  |  |  |  |  |  |  |  |  |  |  |  |  |  |  |
| **ST site** |  |  |  |  |  |  |  |  |  |  |  |  |  |  |  |  |  |  |  |
| **1** | 0.00 | ND | 0.00 | ND | 0.10 | 0.00 | 0.00 | 0.00 | 0.13 | -22.7 | N. D. | -37.0 | -23.4 | 0.21 | -56.1 | 0.03 | -41.2 | 0.04 | -59.0 |
| **3** | 0.04 | -48.0 | 0.02 | -85.6 | 0.07 | 0.01 | 0.01 | 0.00 | 0.16 | -26.2 | -45.5 | -19.4 | -19.2 | 0.18 | -45.6 | 0.06 | -53.7 | 0.02 | -63.8 |
| **5** | 0.06 | -49.7 | 0.05 | -102.5 | 0.04 | 0.01 | 0.01 | 0.00 | 0.05 | -37.7 | -46.7 | -30.6 | -24.2 | 0.10 | -42.7 | 0.02 | -61.5 | 0.02 | -59.0 |
| **7** | 0.05 | -50.7 | 0.08 | -105.2 | 0.04 | 0.01 | 0.01 | 0.00 | 0.03 | -30.6 | -47.5 | -35.5 | -26.8 | 0.08 | -36.1 | 0.01 | ND | 0.01 | ND |
| **9** | 0.05 | -63.8 | 0.08 | -106.1 | 0.03 | 0.01 | 0.01 | 0.00 | 0.02 | -32.9 | -55.8 | -48.9 | -28.5 | 0.08 | -34.8 | 0.01 | ND | 0.01 | ND |
| **12** | 0.05 | -65.5 | 0.09 | -109.6 | 0.03 | 0.01 | 0.01 | 0.00 | 0.02 | -26.1 | -59.5 | -34.6 | -27.3 | 0.01 | ND | 0.01 | ND | 0.01 | ND |
|  |  |  |  |  |  |  |  |  |  |  |  |  |  |  |  |  |  |  |  |
| **Reference site** |  |  |  |  |  |  |  |  |  |  |  |  |  |  |  |  |  |  |  |
| **1** | ND | ND | ND | ND | 0.58 | 0.02 | 0.01 | 0.00 | 0.54 | -19.0 | -40.0 | -28.4 | -27.5 | ND | ND | ND | ND | 0.02 | ND |
| **5** | ND | ND | ND | ND | 0.56 | 0.02 | 0.01 | 0.00 | 0.54 | -27.3 | -45.6 | -31.7 | -27.2 | ND | ND | ND | ND | 0.00 | ND |
| **11** | ND | ND | ND | ND | 0.64 | 0.03 | 0.01 | 0.00 | 0.61 | -27.0 | -38.2 | -22.6 | -28.2 | ND | ND | ND | ND | 0.01 | ND |
| **15** | ND | ND | ND | ND | 0.28 | 0.01 | 0.00 | 0.00 | 0.28 | -26.7 | -40.2 | ND | -30.3 | ND | ND | ND | ND | 0.00 | ND |
| **25** | ND | ND | ND | ND | 0.51 | 0.01 | 0.01 | 0.00 | 0.51 | -23.2 | -41.3 | ND | -26.5 | ND | ND | ND | ND | 0.01 | ND |
| **41** | ND | ND | ND | ND | 0.72 | 0.03 | 0.01 | 0.00 | 0.77 | -21.9 | -43.0 | -23.2 | -23.9 | ND | ND | ND | ND | 0.01 | ND |

ND indicates ‘not determined’.

Table S2. Sequence summary and diversity indices.

| **Core depth (cm)** | **Archaea** | | | **Bacteria** | | |
| --- | --- | --- | --- | --- | --- | --- |
|  | **Reads No.** | **shannon** | **invsimpson** | **Reads No.** | **shannon** | **invsimpson** |
| **DM site** |  |  |  |  |  |  |
| **0** | 26425 | 1.30 | 1.89 | 24612 | 4.69 | 26.29 |
| **1** | 15733 | 1.94 | 3.33 | 33598 | 4.36 | 19.51 |
| **2** | 32348 | 2.75 | 8.21 | 36191 | 4.21 | 17.70 |
| **3** | 36016 | 2.40 | 5.06 | 47345 | 4.11 | 16.23 |
| **4** | 32390 | 2.60 | 6.22 | 44411 | 4.27 | 19.97 |
| **5** | 40056 | 1.82 | 2.71 | 38520 | 4.03 | 14.01 |
| **6** | 44987 | 0.92 | 1.40 | 48774 | 3.15 | 5.50 |
| **7** | 28335 | 0.36 | 1.11 | 18511 | 3.77 | 9.83 |
| **8** | 63274 | 0.26 | 1.07 | 23316 | 3.69 | 9.52 |
| **9** | 54128 | 0.37 | 1.11 | 56241 | 3.89 | 10.94 |
| **10** | 58663 | 0.47 | 1.15 | 36445 | 3.90 | 17.93 |
| **11** | 77592 | 0.41 | 1.11 | 39953 | 3.61 | 12.23 |
| **12** | 44598 | 0.87 | 1.40 | 41731 | 3.78 | 13.45 |
| **13** | 53787 | 1.45 | 2.09 | 36521 | 3.51 | 10.23 |
| **14** | 56888 | 0.95 | 1.46 | 32083 | 2.75 | 6.46 |
| **15** | 54330 | 0.79 | 1.33 | 47485 | 1.98 | 2.98 |
| **16** | 76677 | 0.47 | 1.15 | 63065 | 1.81 | 2.87 |
| **BM site** |  |  |  |  |  |  |
| **0** | N. D | N. D | N. D | 17299 | 4.95 | 39.02 |
| **1** | N. D | N. D | N. D | 16539 | 4.61 | 29.96 |
| **2** | 65506 | 0.73 | 1.35 | 28319 | 4.57 | 27.12 |
| **3** | 43241 | 0.79 | 1.41 | 42456 | 4.53 | 27.27 |
| **4** | 53350 | 0.82 | 1.42 | 20496 | 3.63 | 10.11 |
| **5** | 78511 | 0.50 | 1.18 | 36411 | 3.90 | 10.86 |
| **6** | 53731 | 0.60 | 1.24 | 50541 | 2.91 | 6.25 |
| **7** | 53720 | 0.85 | 1.45 | 30922 | 3.72 | 9.17 |
| **8** | 57837 | 0.72 | 1.34 | 42752 | 3.24 | 8.94 |
| **9** | 33333 | 1.02 | 1.61 | 8070 | 3.63 | 11.96 |
| **10** | 50309 | 0.46 | 1.16 | 15560 | 2.95 | 7.89 |
| **11** | 50744 | 0.90 | 1.53 | 37696 | 3.52 | 11.01 |
| **12** | 61229 | 0.59 | 1.23 | 16626 | 2.95 | 6.28 |
| **13** | 110192 | 0.33 | 1.09 | 48091 | 2.41 | 3.71 |
| **14** | 59775 | 0.35 | 1.12 | 46172 | 2.53 | 4.28 |
| **ST site** |  |  |  |  |  |  |
| **0** | 53595 | 1.14 | 2.27 | 31703 | 6.26 | 167.10 |
| **1** | 8711 | ND | ND | 17776 | 5.97 | 100.60 |
| **2** | 14943 | 2.61 | 7.07 | 31093 | 5.53 | 67.92 |
| **3** | 21304 | 2.63 | 6.69 | 37510 | 5.47 | 66.93 |
| **4** | 23561 | 2.70 | 7.50 | 19399 | 5.31 | 65.44 |
| **5** | 29145 | 2.05 | 3.30 | 34890 | 5.18 | 65.67 |
| **6** | 57862 | 1.39 | 1.94 | 32765 | 4.85 | 39.60 |
| **7** | 69073 | 1.05 | 1.59 | 39684 | 4.51 | 24.25 |
| **8** | 96798 | 0.52 | 1.19 | 39793 | 4.00 | 13.61 |
| **9** | 64466 | 0.61 | 1.24 | 27127 | 3.73 | 10.46 |
| **10** | 81756 | 0.42 | 1.13 | 36660 | 3.38 | 7.90 |
| **11** | 65437 | 0.50 | 1.15 | 37892 | 3.72 | 11.53 |
| **12** | 68786 | 0.29 | 1.08 | 25082 | 3.61 | 11.27 |
| **13** | 71361 | 0.29 | 1.08 | 29515 | 3.43 | 9.93 |
| **14** | 74712 | 0.35 | 1.10 | 46305 | 3.27 | 9.99 |
| **15** | 91436 | 0.27 | 1.07 | 30331 | 3.11 | 8.09 |
| **16** | 122357 | 0.26 | 1.06 | 48043 | 2.80 | 6.97 |
| **17** | 67005 | 0.28 | 1.07 | 39711 | 2.89 | 6.65 |
| **Reference site** |  |  |  |  |  |  |
| **0** | 41921 | 1.12 | 2.13 | 23459 | 6.89 | 331.66 |
| **1** | 35223 | 1.17 | 2.25 | 21928 | 6.77 | 291.09 |
| **2** | 58638 | 1.18 | 2.25 | 31106 | 6.76 | 296.45 |
| **3** | 40573 | 1.32 | 2.52 | 26927 | 6.71 | 285.31 |
| **4** | 36533 | 1.34 | 2.26 | 27294 | 6.75 | 304.76 |
| **5** | 10964 | 1.44 | 2.40 | 27567 | 6.72 | 289.96 |
| **6** | 11303 | 1.59 | 2.49 | 35995 | 6.85 | 340.26 |
| **7** | 26680 | 1.36 | 2.01 | 24851 | 6.76 | 337.02 |
| **8** | 26732 | 1.21 | 1.78 | 40093 | 6.57 | 260.00 |
| **9** | 26206 | 1.80 | 2.59 | 36969 | 6.71 | 301.31 |
| **10** | 36740 | 2.14 | 3.76 | 36404 | 6.51 | 211.19 |
| **11** | 31392 | 1.66 | 2.30 | 30134 | 6.47 | 227.08 |
| **12** | 42173 | 1.44 | 1.90 | 35205 | 6.59 | 259.25 |
| **13** | 10017 | 1.68 | 2.27 | 25018 | 6.51 | 249.97 |
| **14** | 13977 | 1.74 | 2.33 | 26409 | 6.35 | 174.80 |
| **15** | 25397 | 1.84 | 2.58 | 23206 | 6.37 | 187.55 |

ND indicates ‘not determined’.

Table S3. Relative abundance of OTUs of the order *Methylococcales*.

See the Excel file

Table S4. Heat map of major OTUs with taxonomy.

See the Excel file

Table S5. Pearson correlation coefficient between the major OTUs of ANMEs and Deltaproteobacteria.

| **OTU ID** | **DM site** | | | | **BM site** | | | | **ST site** | | | |
| --- | --- | --- | --- | --- | --- | --- | --- | --- | --- | --- | --- | --- |
|  | **A_OTU001** | | **A_OTU008** | | **A_OTU001** | | **A_OTU008** | | **A_OTU001** | | **A_OTU008** | |
|  | **r** | ***p*** | **r** | ***p*** | **r** | ***p*** | **r** | ***p*** | **r** | ***p*** | **r** | ***p*** |
| B_OTU002 | 0.41 | 0.105 | -0.26 | 0.323 | -0.16 | 0.613 | 0.21 | 0.499 | **0.92** | 0.000 | -0.34 | 0.178 |
| B_OTU009 | 0.35 | 0.166 | -0.27 | 0.301 | 0.65 | 0.017 | -0.07 | 0.825 | 0.35 | 0.172 | 0.50 | 0.040 |
| B_OTU011 | 0.33 | 0.200 | -0.24 | 0.353 | -0.20 | 0.522 | 0.03 | 0.926 | **0.91** | 0.000 | -0.20 | 0.442 |
| B_OTU014 | 0.37 | 0.141 | -0.25 | 0.338 | 0.11 | 0.716 | -0.20 | 0.513 | -0.78 | 0.000 | -0.09 | 0.742 |
| B_OTU018 | -0.47 | 0.058 | 0.27 | 0.300 | -0.21 | 0.486 | 0.09 | 0.766 | -0.46 | 0.064 | **0.71** | 0.001 |
| B_OTU024 | 0.22 | 0.393 | -0.18 | 0.496 | -0.23 | 0.451 | -0.07 | 0.821 | 0.34 | 0.175 | 0.18 | 0.498 |
| B_OTU029 | 0.38 | 0.128 | -0.28 | 0.275 | -0.31 | 0.305 | 0.14 | 0.645 | 0.17 | 0.514 | **0.71** | 0.001 |
| B_OTU176 | 0.29 | 0.260 | -0.13 | 0.616 | -0.41 | 0.161 | -0.25 | 0.419 | ND | ND | ND | ND |
| B_OTU010 | -0.76 | 0.000 | 0.10 | 0.705 | -0.17 | 0.574 | -0.01 | 0.962 | -0.81 | 0.000 | 0.44 | 0.077 |
| B_OTU064 | -0.36 | 0.159 | -0.18 | 0.486 | -0.60 | 0.031 | -0.49 | 0.087 | -0.90 | 0.000 | -0.15 | 0.567 |
| B_OTU095 | 0.20 | 0.432 | -0.26 | 0.312 | ND | ND | ND | ND | -0.48 | 0.049 | -0.20 | 0.443 |
| B_OUT119 | -0.66 | 0.004 | -0.09 | 0.725 | -0.07 | 0.823 | -0.10 | 0.756 | -0.72 | 0.001 | -0.29 | 0.262 |

* r indicates the Pearson correlation coefficient and *p* is the *p* value.

Bold indicates significant correlations (*p* < 0.01) between archaeal OTUs and deltaproteobacterial OTUs.

‘ND’ indicates ‘not determined’.


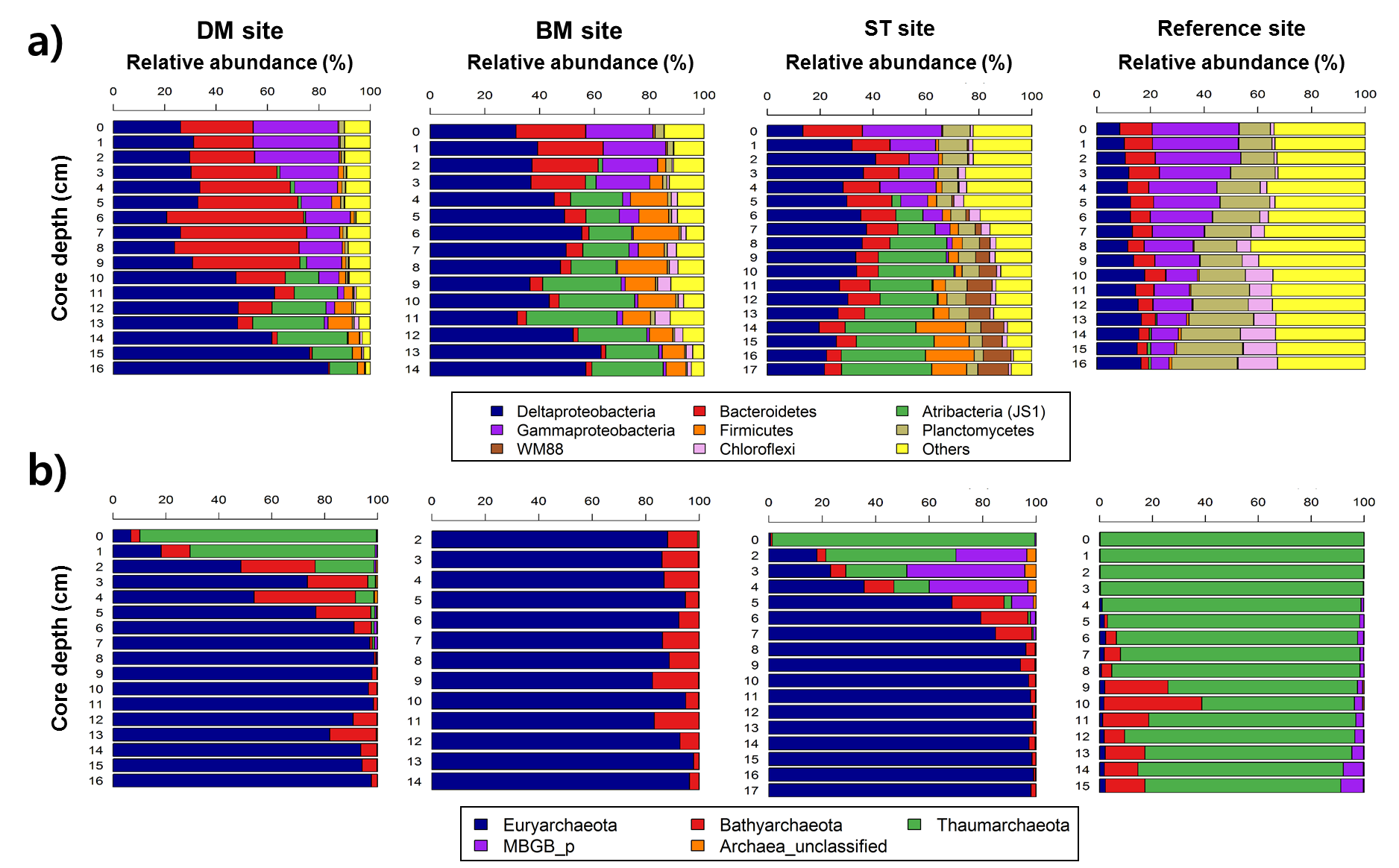


Figure S1. Relative abundance of (a) bacterial taxa at phylum level and class level of Proteobacteria) and (b) archaeal (at class level) along the core depths.


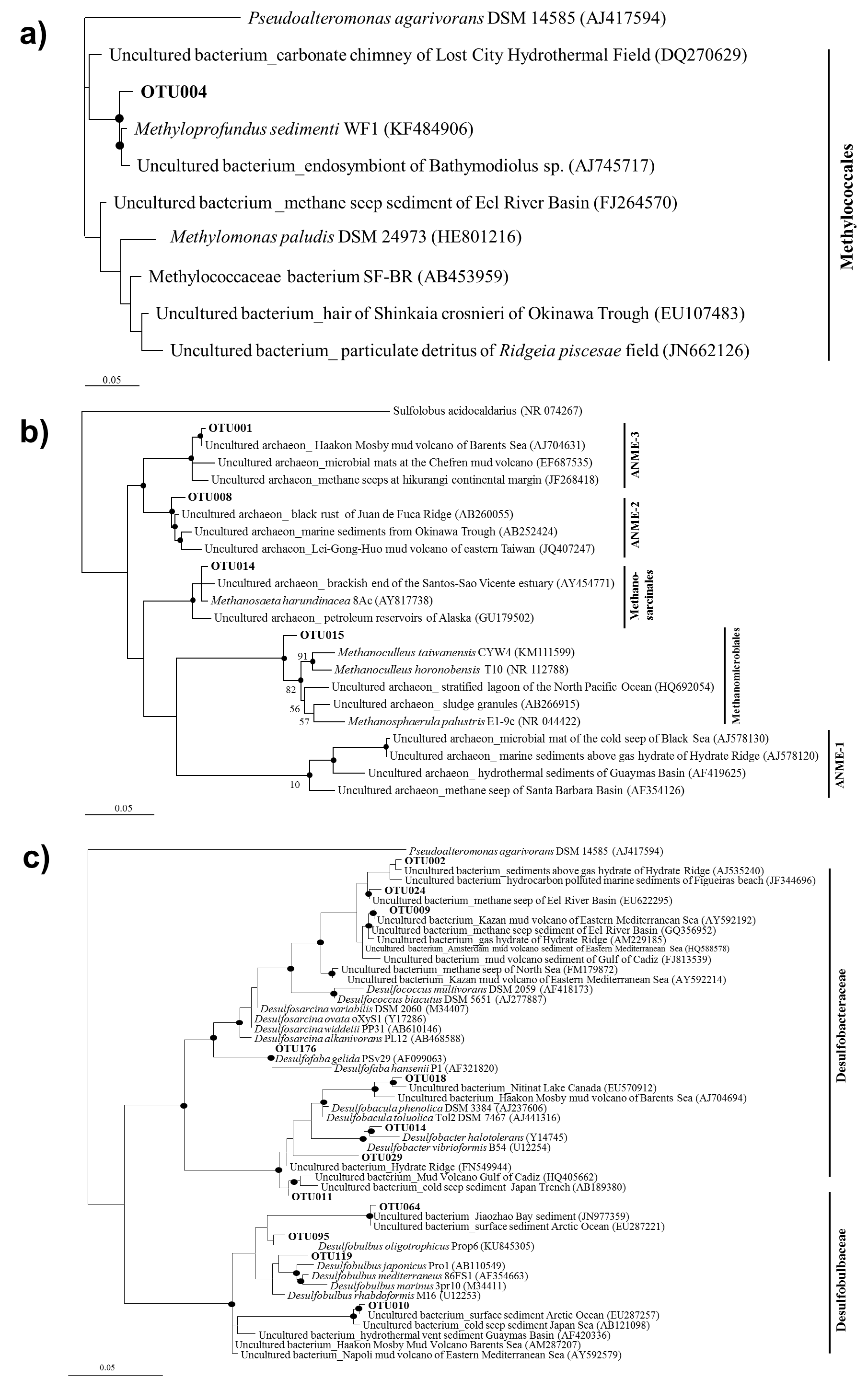


Figure S2. Phylogenetic tree of major OTUs of (a) *Methylococcales,* (b) *Methanomicrobia*, and (c) *Deltaprotepbacteria*. Major OTUs with more than 5 % in relative abundance at each sample were selected and phylogenetic tree was reconstructed by the heuristic search with the maximum likelihood algorithm with the GTR evolutionary model. Filled circles indicate that the corresponding nodes had >70 % bootstrap values based on 1,000 resamplings. Bars indicate 5 nucleotide substitutions per 100 nucleotides.

Sequence S1. 16S rRNA sequences of archaeal OTUs of *Methanomicrobia* with greater than 5 % relative abundance used for the phylogenetic analysis.

>A_OTU001

TCAATCGGAGTCAACGCCGGAAAACTCACCGGGGGCGACAGCAATATGTA

GGTCAGGCTAAAGACCTTACCCGAATCGCTGAGAGGAGGTGCATGGCCGT

CGTCAGTTCGTACTGTGAAGCATCCTGTTAAGTCAGGCAACGAGCGAGAC

CCGTGCCCACTGTTGCCAGCATGCCCTTCGGGGTGATGGGTACTCTGTGG

GGACCGCCGCTGCTAAAGCGGAGGAAGGTGCGGGCTACGGTAGGTCAGTA

TGCCCCGAATCTCCCGGGCTACACGCGGGCTACAATGATTGGGACAATGG

GTCCCTACTCCGAAAGGAGATGGTAATCTCATAAACCCAACCTTAGTTCG

AATTGAGGGCTGTAACTCGCCCTCATGAAGCTGGAATCCGTAGTAATCGC

GTTTCAACATAGCGCGGTGAATACGTCCCTGCCCCTTGCACTCACC

> A_ OTU014

TTAATCGGAGTCAACGCCGGAAATCTCACCGGGGGCGACAGCGATATGAA

GGTCAGGCTGAAGACCTTACCGGATTAGCTGAGAGGTGGTGCATGGCCGT

CGTCAGTTCGTACTGTGAAGCATCCTGTTAAGTCAGGCAACGAGCGAGAC

CCACGTCCACAGTTGCCAGCATACCCTCCGGGGTGATGGGTACACTGTGG

AGACCGCCGCTGCTAAAGCGGAGGAAGGAATGGGCAACGGTAGGTCAGTA

TGCCCCGAATCTCCCGGGCTACACGCGGGCTACAATGGTTGGTACAATGG

GCATCTACCCCGAAAGGGGAAGGAAATCTCTTAAAGCCAATCGTAGTTCG

GATTGAGGGCTGCAACTCGCCCTCATGAAGCTGGAATCCGTAGTAATCGC

GTTTCAACAGAACGCGGTGAATACGTCCCTGCCCCTTGCACACACC

> A_ OTU008

TCAATCGGAGTCAACGCCGGAAAACTCACCGGGTGCGACAGCAACATGTA

GGTCAGGCTGAAGGTCTTACCTGAATCGCTGAGAGGAGGTGCATGGCCGT

CGTCAGTTCGTACTGTGAAGCATCCTGTTAAGTCAGGCAACGAGCGAGAC

CCGCGCCCACAGTTGCCAGCATGTTCTCCGGAACGATGGGCACACTGTGG

GGACCGCCGCTGCTAAAGCGGAGGAAGGAACGGGCTACGGTAGGTCAGTA

TGCCCCGAATCTCCCGGGCTACACGCGGGCTACAATGGTTGGCACAATGG

GCACCTACCCCGAAAGGGGACGGTAATCTCCTAAATCCAACCGTAGTTCG

GATTGAGGGCTGTAACTCGCCCTCATGAAGCTGGAATCCGTAGTAATCGC

GTTTCAACATAGCGCGGTGAATACGTCCCTGCCCCTTGCACTCACC

> A_ OTU015

TTAATTGGAGTCAACGCCGGAAAACTCACCGGATAAGACAGCAGAATGAT

AGCCAGGCTGAAGACCTTGCTTGACCAGCTGAGAGGAGGTGCATGGCCGT

CGTCAGTTCGTACTGTGAAGCATCCTGTTAAGTCAGGCAACGAGCGAGAC

CCACGCCAACAGTTGCCAGCATGTCCTCCGGGATGATGGGGACACTGTTG

GGACCGCCTCTGCTAAAGAGGAGGAAGGAATGGGCAACGGTAGGTCAGCA

TGCCCCGAATTATCCGGGCTACACGCGGGCTACAATGGGCAGGACAATGG

GTATCGACACCGAAAGGTGAAGGAAATCTCCTAAACCTGTCCGTAGTTCG

GATTGTGGGCTGCAACTCGCCCACATGAAGCTGGAATCTGTAGTAATCGC

GTTTCAAAATAGCGCGGTGAATGCGTCCCTGCCCCTTGCACACACC

Sequence S2. 16S rRNA sequences of Deltaproteobacteria with greater than 5 % relative abundance used for the phylogenetic analysis.

> B_OTU002

TGTGAAAGCCCGGGGCTCAACCCCGGAAGTGCATTTGAAACTGAGAGACT

TGAGTATGGGAGAGGGAAGTGGAATTCCTGGTGTAGAGGTGAAATTCGTA

GATATCAGGAGGAACACCGGTGGCGAAGGCGACTTCCTGGACCAATACTG

ACGCTGAGGCGCGAAGGCGTGGGGAGCAAACAGGATTAGATACCCTGGTA

GTCCACGCAGTAAACGGTGAACACTAGGTGTAGCGGGTATTGACCCCTGC

TGTGCCGTAGCTAACGCATTAAGTGTTCCGCCTGGGGAGTACGACCGCAA

GGTTAAAACTCAAAGGAATTGGCGG

> B_OTU009

TGTGAAAGCCCGGGGCTTAACCCCGGAAGTGCATTTGAAACTGAAAAACT

TGAGTATGGGAGAGGGAAGTGGAATTCCTGGTGTAGAGGTGAAATTCGTA

GATATCAGGAGGAACACCGGTGGCGAAGGCGACTTCCTGGACCAATACTG

ACGCTGAGGCGCGAAGGCGTGGGGAGCAAACAGGATTAGATACCCTGGTA

GTCCACGCAGTAAACGATGAACACTAGGTGTAGCGGGTATTGACCCCTGC

TGTGCCGCAGTTAACGCATTAAGTGTTCCGCCTGGGGAGTACGACCGCAA

GGTTAAAACTCAAAGAAATTGACGG

> B_OTU010

TGTGAAAGTCCACGGCTCAACCGTGGAAGTGCATTTGAAACTGGCAAACT

TGAGTACTGGAGGGGGTAGTGGAATTCCCGGTGTAGAGGTGAAATTCGTA

GATATCGGGAGGAATACCGGTGGCGAAGGCGACTACCTGGCCAGATACTG

ACACTGAGGTGCGAAAGCGTGGGGAGCAAACAGGATTAGATACCCTGGTA

GTCCACGCCGTAAACGATGTCAACTAGGTGTTGGGATGGTTAATCGTCTC

ATTGCCGCAGCTAACGCATTAAGTTGACCGCCTGGGGAGTACGGTCGCAA

GATTAAAACTCAAAGAAATTGACGG

> B_OTU011

TGTGAAAGCCCGGGGCTCAACCCTGGAAGTGCATTTGAAACAGCAAGACT

TGAGTACGGGAGAGGAAAGCGGAATTCCTGGTGTAGAGGTGAAATTCGTA

GATATCAGGAGGAACACCGATGGCGAAGGCAGCTTTCTGGACCGATACTG

ACGCTGAGGCGCGAAGGCGTGGGTAGCGAACAGGATTAGATACCCTGGTA

GTCCACGCAGTAAACGATGTTCACTAGGTGTAGCGGGTATTAAAATCTGC

TGTGCCGGAGCTAACGCATTAAGTGAACCGCCTGGGGAGTACGGTCGCAA

GACTAAAACTCAAAGAAATTGACGG

> B_OTU014

TGTGAAAGCCCGGGGCTTAACCCCGGAAGTGCACTTGAAACAGCAAGACT

TGAATACGGGAGAGGAAAGCGGAATTCCTGGTGTAGAGGTGAAATTCGTA

GATATCAGGAGGAACACCGATGGCGAAGGCAGCTTTCTGGACCGATATTG

ACGCTGAGGCGCGAAGGCGTGGGTAGCGAACGGGATTAGATACCCCGGTA

GTCCACGCAGTAAACGTTGTACACTCGGTGTGGCGGATATTAAAATCTGC

TGTGCCTAAGCTAACGCATTAAGTGTACCGCCTGGGAAGTACGGTCGCAA

GACTAAAACTCAAAGGAATTGGCGG

> B_OTU018

TGTGAAAGCCCAGGGCTCAACCCTGGACGTGCATTTGAAACAGTAAGACT

TGAGTACTGGAGAGGAAAGCGGAATTCCTGGTGTAGAGGTGAAATTCGTA

GATATCAGGAGGAACACCGATGGCGAAGGCAGCTTTCTGGACAGAAACTG

ACGCTGATGCGCGAAGGCGTGGGTAGCGAACAGGATTAGATACCCTGGTA

GTCCACGCAGTAAACGATGTACACTAGGTGTAGGGGATATTAAAATTTTC

TGTGCCGCAGCTAACGCATTAAGTGTACCGCCTGGGGAGTACGGTCGCAA

GACTAAAACTCAAAGAAATTGACGG

> B_OTU024

TGTGAAAGCCCGGGGCTCAACCCCGGAAGTGCATTTGAAACTAATGGACT

TGAGTATGGGAGAGGGAAGTGGAATTCCTGGTGTAGAGGTGAAATTCGTA

GATATCAGGAGGAACACCGGTGGCGAAGGCGACTTCCTGGACCAATACTG

ACGCTGAGGCGCGAAGGCGTGGGGAGCAAACAGGATTAGATACCCTGGTA

GTCCACGCAGTAAACGGTGAACACTAGGTGTAGCGGGTATTGACCCCTGC

TGTGCCGCAGTTAACGCATTAAGTGTTCCGCCTGGGGAGTACGGCCGCAA

GGTTAAAACTCAAAGAAATTGACGG

> B_OTU029

TGTGAAAGCCCGGGGCTCAACCCTGGAAGTGCATTTGAAACAGCAAGACT

TGAGTACGGGAGAGGAGAGCGGAATTCCTGGTGTAGAGGTGAAATTCGTA

GATATCAGGAGGAACACCGATGGCGAAGGCAGCTCTCTGGACCGATACTG

ACGCTGAGGCGCGAAGGTGTGGGTAGCGAACAGGATTAGATACCCTGGTA

GTCCACACAGTAAACGTTGTTCACTCGGTGTAGCGGATATTAAAATCTGC

TGTGCCCAAGCTAACGTGGTAAGTGAACCGCCTGGGGAGTACGGTCGCAA

GACTAAAACTCAAATAAATTGACGG

> B_OTU095

TGTGAAAGCCCACGGCTTAACCGTGGAAGTGCATTTGAAACTGTCAGACT

TGAGTACCAGAGGGGAAAGTGGAATTCCCGGTGTAGAGGTGAAATTCGTA

GATATCGGGAGGAATACCGGTGGCGAAGGCGACTTTCTGGCTGGATACTG

ACGCTGAGGCGCGAAAGCGTGGGGAGCAAACAGGATTAGATACCCTGGTA

GTCCACGCTGTAAACGATGTGAACTAGGTGTAGGGGGTGTTGATCCCTTC

TGTGCCGCAGCTAACGCATTAAGTTCACCGCCTGGGGAGTACGGTCGCAA

GATTAAAACTCAAAGAAATTGACGG

> B_OTU119

TGTGAAAGCCCACGGCTCAACCGTGGAAGTGCATTTGAAACTGCTTGGCT

TGAGTATCAGAGGGGAAAGTGGAATTCCCGGTGTAGAGGTGAAATTCGTA

GATATCGGGAGGAATACCGGTGGCGAAGGCGACTTTCTGGCTGAATACTG

ACGCTGAGGCGCGAAAGCGTGGGGAGCAAACAGGATTAGATACCCTGGTA

GTCCACGCCGTAAACGATGTCAACTAGGTGCAGGGGGTGTTGATCCCCTC

TGTGTCGCAGCTAACGCATTAAGTTGACCGCCTGGGGAGTACGGTCGCAA

GATTAAAACTCAAAGGAATTGACGG

> B_OTU176

TGTGAAATCCCGGGGCTCAACCCCGGAAGTGCATTTGAAACCCCGAGACT

TGAGTATGGGAGAGGGAAGTGGAATTCCAGGTGTAGAGGTGAAATTCGTA

GATATCTGGAGGAACACCGGTGGCGAAGGCGACTTCCTGGACCAATACTG

ACGCTGAGGTGCGAAGGCGTGGGGAGCAAACAGGATTAGATACCCTGGTA

GTCCACGCAGTAAACGTTGTTCACTAGGTGTAGCGGGTATTGACTCCTGC

TGTGCCGCAGCTAACGCATTAAGTGAACCGCCTGGGGAGTACGATCGCAA

GATTAAAACTCAAAGAAATTGACGG

Sequence S3. 16S rRNA sequences of bacterial OTUs of *Methylococcales* of Gammaprotoebacteria with greater than 5 % relative abundance used for phylogenetic the analysis.

>B_OTU004

GTGCCAGCCGCCGCGGTAATACGGAGGGTGCAAGCGTTAATCGGAATTAC

TGGGCGTAAAGCGTTCGTAGGTGGTTAGTTAAGTTAGATGTGAAAGCCCC

GGGCTTAACCTGGGAACTGCATTTAAAACTGGCTAACTAGAGTTTAGGAG

AGGGAAGTGGAATTTCAGGTGTAGCAGTGAAATGCGTAGAGATCTGAAGG

AACACCAGTGGCGAAGGCGACTTCCTGGACTAAAACTGACGCTGAGGAAC

GAAAGCGTGGGTAGCAAACGGGATTAGATACCCCGGTAGTCCACGCCGTA

AACGATGTCAACTAGCCGTTGGGCCTATTTATAGGCTTAGTGGCGGAGCT

AACGTATTAAGTTGACCGCCTGGGGAGTACGGCCGCAAGGTTAAAACTCA

AAGGAATTGGCGG
